# Comparison of machine learning models based on multi-parametric magnetic resonance imaging and ultrasound videos for the prediction of prostate cancer

**DOI:** 10.3389/fonc.2023.1157949

**Published:** 2023-05-16

**Authors:** Xiaoyang Qi, Kai Wang, Bojian Feng, Xingbo Sun, Jie Yang, Zhengbiao Hu, Maoliang Zhang, Cheng Lv, Liyuan Jin, Lingyan Zhou, Zhengping Wang, Jincao Yao

**Affiliations:** ^1^ Department of Ultrasound, The Affiliated Dongyang Hospital of Wenzhou Medical University, Dongyang, Zhejiang, China; ^2^ Department of Ultrasound, The Cancer Hospital of the University of Chinese Academy of Sciences (Zhejiang Cancer Hospital), Hangzhou, Zhejiang, China; ^3^ Institute of Basic Medicine and Cancer (IBMC), Chinese Academy of Sciences, Hangzhou, Zhejiang, China; ^4^ Key Laboratory of Head & Neck Cancer Translational Research of Zhejiang Province, The Cancer Hospital of the University of Chinese Academy of Sciences (Zhejiang Cancer Hospital) Zhejiang Provincial Research Center for Cancer Intelligent Diagnosis and Molecular Technology, Hangzhou, Zhejiang, China

**Keywords:** radiomics, prostate cancer, machine learning, ultrasound, magnetic resonance imaging, support vector machines

## Abstract

**Objective:**

To establish machine learning (ML) prediction models for prostate cancer (PCa) using transrectal ultrasound videos and multi-parametric magnetic resonance imaging (mpMRI) and compare their diagnostic performance.

**Materials and methods:**

We systematically collated the data of 383 patients, including 187 with PCa and 196 with benign lesions. Of them, 307 patients (150 with PCa and 157 with benign lesions) were randomly selected to train and validate the ML models, 76 patients were used as test set. B-Ultrasound videos (BUS), mpMRI T2 sequence (T2), and ADC sequence (ADC) were obtained from all patients. We extracted 851 features of each patient in the BUS, T2, and ADC groups and used a t-test, the Mann–Whitney U test, and LASSO regression to screen the features. Support vector machine (SVM), random forest (RF), adaptive boosting (ADB), and gradient boosting machine (GBM) models were used to establish radiomics models. In addition, we fused the features screened *via* LASSO regression from three groups as new features and rebuilt ML models. The performance of the ML models in diagnosing PCa in the BUS, T2, ADC, and fusion groups was compared using the area under the ROC curve (AUC), sensitivity, specificity, and accuracy.

**Results:**

In the test cohort, the AUC of each model in the ADC group was higher than that of in the.BUS and T2 groups. Among the models, the RF model had the best diagnostic performance, with an AUC of 0.85, sensitivity of 0.78 (0.61–0.89), specificity of 0.84 (0.69–0.94), and accuracy of 0.83 (0.66–0.93). The SVM model in both the BUS and T2 groups performed best. Based on the features screened in the BUS, T2, and ADC groups fused to construct the models, the SVM model was found to perform best, with an AUC of 0.87, sensitivity of 0.73 (0.56–0.86), specificity of 0.79 (0.63–0.90), and accuracy of 0.77 (0.59–0.89). The difference in the results was statistically significant (*p*<0.05).

**Conclusion:**

The ML prediction models had a good diagnostic ability for PCa. Among them, the SVM model in the fusion group showed the best performance in diagnosing PCa. These prediction models can help radiologists make better diagnoses.

## 1 Introduction

Prostate cancer (PCa) is the most common malignant tumor in the genitourinary system of elderly men, ranking second in incidence of male malignant tumors (1). Although the incidence of PCa in China is much lower than in developed Western countries, it is increasing more rapidly (2.6% per year) than in developed Western countries with the “Westernization” of lifestyle and the rapid aging of the population (2). PCa occurs in the prostate epithelium with an insidious onset and a lack of typical clinical manifestations, so many patients are diagnosed at an advanced stage, when it is often accompanied by bone metastases (3). Therefore, early detection and diagnosis are key factors to determine treatment outcomes. Prostate-specific antigen (PSA) level detection, digital rectal examination, and transrectal ultrasound (TRUS)-guided prostate biopsy are the most commonly used methods for screening PCa in clinical practice (4, 5), despite some degree of overdiagnosis (6). Philip et al. (7) found that the overall cancer detection rate in men with PSA levels exceeding 10 ng/ml was 43%. In a study by Ozorak (8) et al., 56% of patients had PSA levels of 10.1–20 ng/ml and the cancer detection rate in patients with PSA levels > 20 ng/ml was 82.3%. As a screening indicator for PCa, PSA is susceptible to other factors such as urinary retention and prostatitis.

Currently, TRUS has become common for screening PCa because of its convenience and affordability, and the absence of significant contraindications. In TRUS, PCa appears as a hypoechoic area with indistinct margins (9), but many benign prostate diseases also present with similar ultrasound presentation, and the accuracy of the examination depends on both the performance of the examination equipment and also the experience and technique of the examining physician, which is somewhat subjective, resulting in low accuracy for PCa diagnosis. Multi-parametric MRI (mpMRI) achieves good soft tissue resolution, and scholars researchers widely agree that MRI signal changes of prostate tumor lesions are characteristic, which can improve the diagnostic accuracy and help reduce overdetection and overtreatment in clinical practice (10). Clinically significant PCa diagnosis based on Prostate Imaging Reporting & Data System (PI-RADS) relies heavily on the clinical experience of radiologists; the false-positive rate of mpMRI is high and the interobserver agreement is poor (11). Magnetic resonance imaging (MRI) and ultrasound can noninvasively provide morphological and some functional tumor information but cannot provide a deeper analysis of the heterogeneous characteristics of tumors.

Radiomics can extract high-throughput, quantitative image features from medical images and mine information related to tumor pathophysiology (12). Through machine learning (ML), screening and classification of a large number of image phenotypic features can be performed to evaluate tissues noninvasively and quantitatively, which can allow more accurate diagnoses and differential diagnoses of lesions compared with traditional imaging examinations. Therefore, we used TRUS videos and mpMRI of the prostate to establish ML prediction models for PCa. The purpose of this study is to compare TRUS videos and mpMRI and discover their respective advantages and disadvantages to facilitate the selection of the best prediction model to improve the detection rate of PCa and avoid transitional diagnoses.

## 2 Materials and methods

### 2.1 Case collection and grouping

All ultrasound videos and mpMRI were collected from Dongyang Affiliated Hospital of Wenzhou Medical University. We recorded a total of 519 cases from July 2021 to June 2022, including 245 cases of PCa and 274 cases of benign prostate lesions.

**Figure 1:**
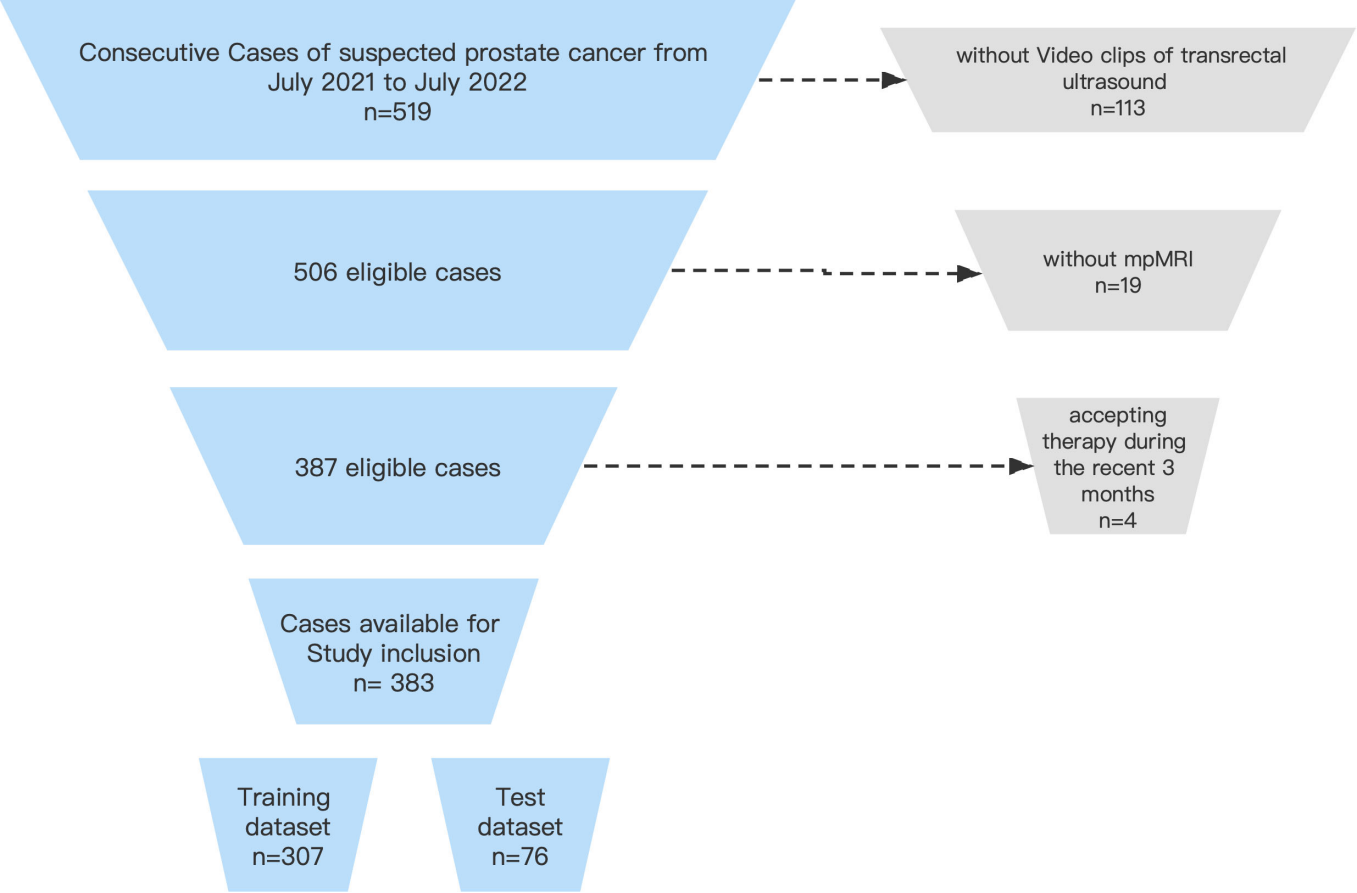
Flowchart of inclusion and exclusion of the study population.

The inclusion criteria were as follows: (i) prostate-occupying lesions detected on rectal finger examination, TRUS, or MRI; (ii) pathological results obtained from prostate puncture biopsy or surgery; and (iii) no relevant treatment received within 3 months. The flowchart of inclusion and exclusion of the study population is shown in **Figure 1**.

Finally, a total of 383 cases were included in this study: 187 cases of PCa and 196 cases of benign lesions. We randomized all cases into a training cohort (307 cases, including 157 cases of benign lesions and 150 cases of malignant lesions) and a test cohort (76 cases, including 39 cases of benign lesions and 37 cases of malignant lesions) at a ratio of 8:2, and then randomly selected 20% of the cases from the training cohort as the validation set before training. The training cohort was used for feature screening and model construction and validation, while the test cohort was used only for model testing.

### 2.2 Ultrasound video and mpMRI acquisition

We collected all prostate-related ultrasound data using an Esaote MyLab Class C diagnostic ultrasound machine (Esaote, Genoa, Italy) with a TRT33 transrectal biplane probe (frequency: 3–13 MHz). Four sonographers with more than 10 years of experience performed TRUS. The patients lay flat on the treatment bed in a lithotomy position, and the sonographers placed the condomed TRT33 probe transanally into the patients’ rectum. Thereafter, the sonographers viewed the prostate pattern on the ultrasound screen, adjusted the probe to the optimal depth, manipulated the probe to scan the entire transverse section of the prostate from top to bottom, and stored a 10-second motion video.

All mpMRI examinations were conducted using a 3.0T Siemens magnetic atomic antenna MRI scanner (Erlangen, Germany), from which we collected all T2 and ADC sequence images containing the prostate.

Three types of image information were obtained from the cases: B-Ultrasound videos, mpMRI T2 sequences, and ADC sequences. We divided the data into three groups according to the image source: BUS, T2, and ADC groups.

### 2.3 Manual annotation

We used a 3D slicer software (v.5.03) for manual annotation, and all three sets of images were outlined with the entire prostate as the region of interest by three ultrasonographers with more than 5 years of experience in TRUS diagnosis. We obscured identifying information, such as the name for each case, named the cases by number, and randomly disorganized them to ensure that the three sonographers could not obtain other relevant information before outlining.

Intra- and inter-class correlation coefficients (ICCs) were used to evaluate the reproducibility of imaging histology feature extraction. Only the manually labeled features with ICCs of ≥0.8, which indicated good reproducibility in the other feature selection process, were included.

### 2.4 Feature extraction

We used the SlicerRadiomics [v.aa418a5 (2022-07-08)] extension package in the 3D Slicer software to extract the imaging histology features (13). For each training cohort in the three groups, we extracted 851 features from each case, including 14 shape features, 18 first-order features, 24 GLCM features, 14 gray level dependency matrix (GLDM) features, 16 GLRM features, 16 gray level size zone matrix (GLSZM) features, 5 NGDM features, and 744 wavelet features. We used the computer programming language Python (v.3.8.8) for subsequent feature screening and model construction, validation, and testing. First, the Levene chi-square test was performed for all features, and the two independent-samples t-test (features with chi-square) and the Mann–Whitney U test (features with chi-square) were used to test for differences between the benign and malignant groups, retaining features that were significantly different (*p*<0.05). Second, we added labels to the data of each case according to the pathological findings, with 0 representing benign and 1 malignant. Finally, the remaining features were screened using LASSO regression, and 10-fold cross-validation was repeated 1,000,000 times in the training cohort to obtain the optimal values of Λ and finally the most valuable features.

### 2.5 ML models

We used the scikit-learn 0.24.2 extension package in Python for the construction of the ML models (14) and randomly divided the training cohort into a training set including 246 cases and an internal validation set including 62 cases at a ratio of 8:2. We used the support vector machine (SVM) model with Gaussian kernel, random forest (RF) model, adaptive boosting (ADB) model, and gradient boosting machine (GBM) model to classify the features in the training set. The best ML model was determined *via* grid search and cross-validation (GridSearchCV) to find the parameters with the highest accuracy for each model in the validation queue.

### 2.6 Optimization model

First, we fused the features filtered *via* LASSO regression from the BUS, T2, and ADC groups as new features and applied the SVM, RF, ADB, and GBM models to the validation and test cohorts to obtain the ROC curves.

Second, we asked three SRs (all with more than 5 years of MRI experience) to diagnose the cases based on the prostate MRI in the validation cohort and selected the diagnoses *via* a minority–majority approach to obtain the AUC.

Third, we combined the diagnostic results of each ML model with the diagnostic results from the senior physicians for voting, with the aim of obtaining a diagnostic result better than that obtained from each model alone.

### 2.7 Statistical analysis

**Figure 2:**
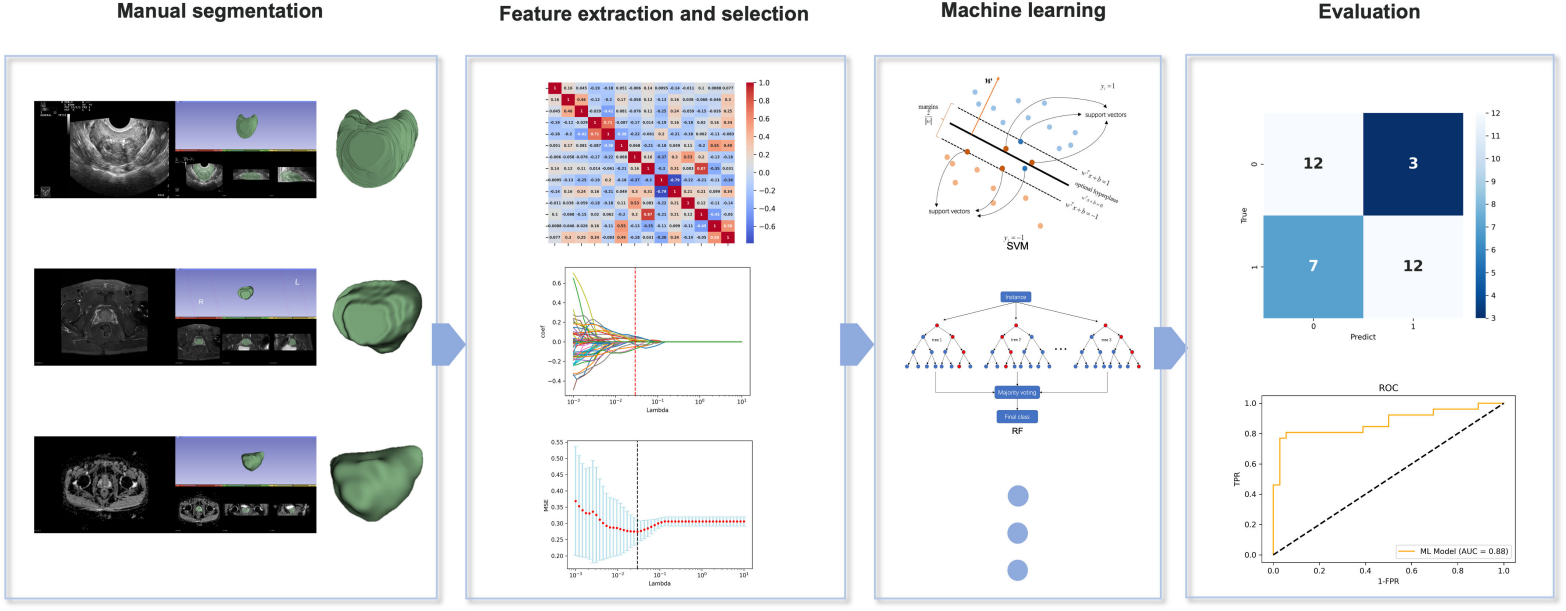
Overall flowchart of the study, including manual segmentation, feature extraction, feature selection, machine learning, and evaluation.

Python and SPSS (v.25.0) were used for the statistical analysis. The chi-square test was used to compare the categorical variables; the continuous variables were presented as means (standard deviations) and categorical variables as numbers (percentages). We also evaluated the superiority of the four ML models in diagnosing PCa in the BUS, T2, and ADC groups based on the AUC, sensitivity, specificity, and accuracy. A *p*-value of <0.05 indicated a statistically significant difference. The overall flowchart of the study is shown in **Figure 2**.

## 3 Results

### 3.1 Clinical case characteristics

The mean age of the training and test cohorts was 71.24 and 71.46 years, respectively, and the mean PSA level was 33.55 and 32.49 ng/mL, respectively (*p*>0.05). These findings indicated no significant differences in age and the PSA level between the two cohorts and no significant selection bias.

In the training cohort, there were 157 (51.1%) cases of benign prostate lesions, including 133 (43.4%) cases of prostate hyperplasia, 19 (6.2%) cases of prostate hyperplasia with prostatitis, 3 (1.0%) cases of prostate hyperplasia with basal cell hyperplasia, and 2 (0.6%) cases of prostate hyperplasia with low-grade intraepithelial neoplasia. Further, there were 150 (48.9%) cases of PCa, of which the Gleason score (GS) was 6 in 59 (19.2%), 7 in 53 (17.3%), 8 in 24 (7.8%), and >8 in 14 (4.6%) cases.

In the test cohort, there were 39 (51.3%) cases of benign prostate lesions, including 33 (43.4) cases of prostatic hyperplasia, 3 (4.0%) cases of prostatic hyperplasia with prostatitis, 2 (2.6%) cases of prostatic hyperplasia with basal cell hyperplasia, and 1 (1.3%) case of prostatic hyperplasia with low-grade intraepithelial neoplasia. Moreover, there were 37 (48.7%) cases of PCa, of which the GS was 6 in 12 (15.8%), 7 in 17 (22.3%), 8 in 4 (5.3%), and >8 in 4 (5.3%) cases.

The clinicopathological characteristics of the training and test cohorts of the study are shown in **Table 1**.

**Table 1:** Clinicopathological characteristics of the training and test cohorts.

	Training set	Test set	P value
NO. of studies	307	76	
Age(y)*	71.2378+-7.94335	71.4605+-8.87610	0.831
PSA(ng/mL)*	33.5454+-126.63170	32.4897+-133.63170	0.949
Pathology			
No.of Benign(-)(%)	157 (51.1%)	39 (51.3%)	
BPH	133 (43.3%)	33 (43.4%)	
BPH & prostatitis	19 (6.2%)	3 (4.0%)	
BPH & BCH	3 (1.0%)	2 (2.6%)	
BPH & LGIN	2 (0.6%)	1 (1.3%)	
No.of Pca(+)(%)	150 (48.9%)	37 (48.7%)	
GS6	59 (19.2%)	12 (15.8%)	
GS7	53 (17.3%)	17 (22.3%)	
GS8	24 (7.8%)	4 (5.3%)	
GS>8	14 (4.6%)	4 (5.3%)	

^*^Data are expressed as means ± standard deviations.

BPH, benign prostatic hyperplasia; BCH, basal cell hyperplasia; LGIN, low-grade intraepithelial neoplasia. *p*<0.05 indicates significant differences in the clinicopathological features in the validation and test sets.

### 3.2 Feature screening

**Figure 3:**
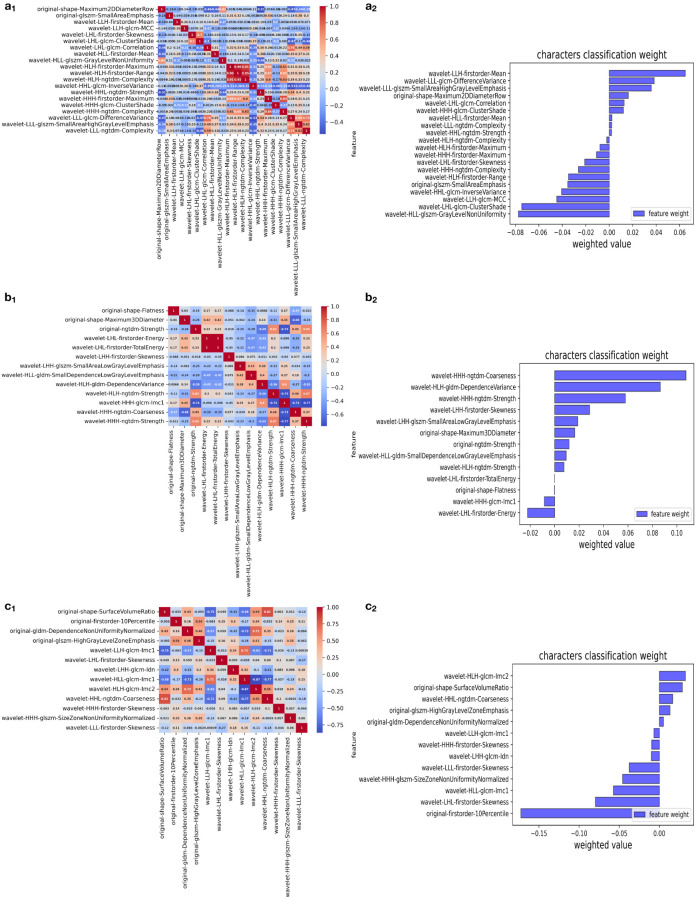
Selection of significant features in the training set and definition of the linear predictor. (a_1_, b_1_, c_1_) Spearman’s correlation coefficients were calculated for the selected features of the BUS, T2, and ADC groups. (a_2_, b_2_, c_2_) Character classification weight of the features of the BUS, T2, and ADC groups.

We extracted 851 features from the image data of each case in the BUS, T2, and ADC groups and screened them using a *t*-test and the Mann-Whitney *U* test, depending on the outcome of Levene’s test: if *p* > 0.05, the two groups of samples were compared by an independent two-sample *t*-test (H_1_: the means of the sample distributions differ); otherwise, the Mann-Whitney *U* test was used with continuity correction and H_1_: the distributions of the two data sets differ. After LASSO regression and dimensionality reduction screening, 20, 13, and 13 features were left in the BUS, T2, and ADC groups, respectively (**Table 2**). In the LASSO regression, we used 10-fold cross-validation to generate the optimal penalty coefficient λ, which was 0.016, 0.024, and 0.024 for the three groups, respectively. The performance of the three groups of screened features is shown in **Figure 3**.

**Table 2:** The subset of radiomics features ultimately selected by the LASSO algorithm.

Feature	Image type	Feature class	Feature name	LASSO coefficients
BUS				
1	Original	Shape	Maximum2DDiameterRow	0.016278
2	Original	glszm	SmallAreaEmphasis	-0.035462
3	wavelet-LLH	firstorder	Mean	0.064800
4	wavelet-LLH	glcm	MCC	-0.447730
5	wavelet-LHL	firstorder	Skewness	-0.021033
6	wavelet-LHL	glcm	ClusterShade	-0.074558
7	wavelet-LHL	glcm	Correlation	0.012700
8	wavelet-HLL	firstorder	Mean	0.002197
9	wavelet-HLL	glszm	GrayLevelNonUniformity	-0.077523
10	wavelet-HLH	firstorder	Maximum	-0.008058
11	wavelet-HLH	firstorder	Range	-0.034742
12	wavelet-HLH	ngtdm	Complexity	-0.002279
13	wavelet-HHL	glcm	InverseVariance	-0.040718
14	wavelet-HHL	ngtdm	Strength	0.001840
15	wavelet-HHH	firstorder	Maximum	-0.010937
16	wavelet-HHH	glcm	ClusterShade	0.012013
17	wavelet-HHH	ngtdm	Complexity	-0.026380
18	wavelet-LLL	glcm	DifferenceVariance	0.038269
19	wavelet-LLL	glszm	SmallAreaHighGrayLevelEmphasis	0.035822
20	wavelet-LLL	ngtdm	Complexity	0.002062
T2				
1	Original	Shape	Flatness	-0.000019
2	Original	Shape	Maximum3Ddiameter	0.001639
3	Original	ngtdm	Strength	0.001174
4	wavelet-LHL	firstorder	Energy	-0.002221
5	wavelet-LHL	firstorder	TotalEnergy	0.000001
6	wavelet-LHH	firstorder	Skewness	0.002864
7	wavelet-LHH	glszm	SmallAreaHighGrayLevelEmphasis	0.001878
8	wavelet-HLL	gldm	SmallDependenceLowGrayLevelEmphasis	0.000943
9	wavelet-HLH	gldm	DifferenceVariance	0.008648
10	wavelet-HLH	ngtdm	Strength	0.000759
11	wavelet-HHH	glcm	Imc1	-0.000859
12	wavelet-HHH	ngtdm	Coarseness	0.010744
13	wavelet-HHH	ngtdm	Strength	0.005782
ADC				
1	Original	Shape	SurfaceVolumeRatio	0.028674
2	Original	firstorder	10Percentile	-0.172593
3	Original	gldm	DependenceNonUniformityNromalized	0.005133
4	Original	glszm	HighGrayLevelZoneEmphasis	0.013405
5	wavelet-LLH	glcm	Imc1	-0.006757
6	wavelet-LHL	firstorder	Skewness	-0.079605
7	wavelet-LHH	glcm	Idn	-0.009877
8	wavelet-HLL	glcm	Imc1	-0.057371
9	wavelet-HLH	glcm	Imc2	0.032562
10	wavelet-HHL	ngtdm	Coarseness	0.016633
11	wavelet-HHH	firstorder	Skewness	-0.009469
12	wavelet-HHH	glszm	SizeZoneNonUniformityNormalized	-0.045828
13	wavelet-LLL	firstorder	Skewness	-0.037563

### 3.3 Model evaluation

**Figure 4:**
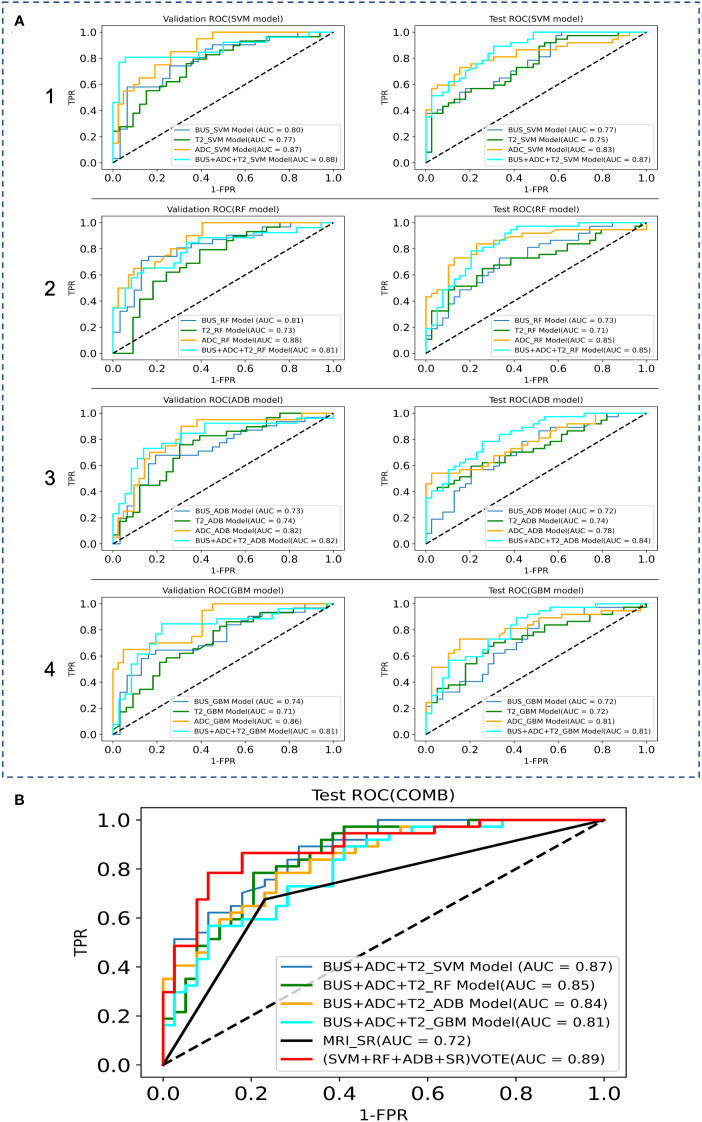
Comparison of the ROC curves between the ML models and MRI data in the validation and test sets, where TPR denotes True Positive Rate and FPR denotes False Positive Rate. **(A)** ROC curves of the internal validation and test sets; 1 SVM models, 2 RF models, 3 ADB models, 4 GBM models. **(B)** ROC curves of the combination model.

The AUC, sensitivity, specificity, and accuracy of the SVM, RF, ADB, and GBM models in the test cohort among the BUS, T2, and ADC groups are shown in **Table 3**, while the ROC curves are displayed in **Figure 4**. The AUC in the ADC group was higher than that in the other two groups. The RF model showed the best diagnostic performance, with an AUC of 0.85, sensitivity of 0.78 (0.61–0.89), specificity of 0.84 (0.69–0.94), and accuracy of 0.83 (0.66–0.93), indicating that the ML model based on ADC performed best. The SVM model in both the BUS and T2 groups showed the best performance. The sensitivity of the model in the BUS group was higher than that in the T2 group, while the specificity and accuracy in the BUS group were lower than those in the T2 group. However, the AUC of the SVM and RF models in the BUS group was higher than that in the T2 group, indicating that the ML models in these two groups had their own advantages and disadvantages.

**Table 3:** Diagnostic performance of machine learning model and MRI on a per-lesion basis.

Model and Dataset	Sensitivity(95%CI)	Specificity(95%CI)	(95%CI)	AUC	P Value	Kappa
BUS						
**SVM Model**	0.57(0.40-0.72)	0.74(0.58-0.86)	0.68(0.49-0.83)	0.77	0.0058	0.312
**RF Model**	0.59(0.42-0.75)	0.72(0.55-0.84)	0.67(0.48-0.81)	0.73	0.006	0.313
**ADB Model**	0.65(0.47-0.79)	0.67(0.50-0.80)	0.65(0.47-0.79)	0.72	0.006	0.315
**GBM Model**	0.65(0.47-0.79)	0.67(0.50-0.80)	0.65(0.47-0.79)	0.72	0.006	0.315
T2						
**SVM Model**	0.46(0.30-0.63)	0.87(0.72-0.95)	0.77(0.55-0.91)	0.75	0.001	0.335
**RF Model**	0.51(0.35-0.68)	0.79(0.63-0.90)	0.70(0.50-0.86)	0.71	0.005	0.331
**ADB Model**	0.57(0.40-0.72)	0.79(0.63-0.90)	0.72(0.53-0.87)	0.74	0.001	0.364
**GBM Model**	0.57(0.40-0.72)	0.79(0.63-0.90)	0.72(0.53-0.87)	0.72	0.001	0.364
ADC						
**SVM Model**	0.73(0.56-0.86)	0.82(0.66-0.92)	0.79(0.62-0.91)	0.83	0.000001	0.551
**RF Model**	**0.78(0.61-0.89)**	0.84(0.69-0.94)	**0.83(0.66-0.93)**	0.85	0.000000	0.631
**ADB Model**	0.59(0.42-0.75)	0.74(0.57-0.86)	0.69(0.50-0.83)	0.78	0.000089	0.420
**GBM Model**	0.59(0.42-0.75)	0.74(0.57-0.86)	0.69(0.50-0.83)	0.81	0.000089	0.420
**MRI-SR**	0.68(0.50-0.81)	0.77(0.60-0.88)	0.74(0.55-0.86)	0.72	0.000097	0.446
BUS+T2+ADC						
**SVM Model**	0.73(0.56-0.86)	0.79(0.63-0.90)	0.77(0.59-0.89)	0.87	0.000005	0.525
**RF Model**	0.76(0.58-0.88)	0.69(0.52-0.82)	0.70(0.53-0.83)	0.85	0.000089	0.448
**ADB Model**	0.59(0.42-0.75)	0.82(0.66-0.92)	0.76(0.56-0.89)	0.84	0.000196	0.417
**GBM Model**	0.65(0.47-0.79)	0.72(0.55-0.84)	0.69(0.51-0.83)	0.81	0.001	0.367
**VOTE**	0.76(0.58-0.87)	**0.90(0.74-0.97)**	0.80(0.64-0.90)	**0.89**	0.000000	**0.656**

SVM model, support vector machine model; RF model, random forest model; ADB model, Adaboosting model; GBM model, gradient boosting machine model; MRI-SR, senior radiologists’ (more than 5 years of experience) diagnosis based on MRI, VOTE, the vote from SVM model, RF model, ADB model and MRI-SR. p<0.05 indicates a significant difference in the discrimination of the SVM model and MRI diagnosis.

We fused the features filtered from the BUS, T2, and ADC groups and then used GridSearchCV to derive the best parameters in the SVM, RF, ADB, and GBM models. After the construction of the four models, classification prediction was then successively performed in the internal validation and test sets. The SVM model performed best, with an AUC of 0.87, sensitivity of 0.73 (0.56–0.86), specificity of 0.79 (0.63–0.90), and accuracy of 0.77 (0.59–0.89).

The results obtained from the chi-square test in each group were significantly different (*p*<0.05).

### 3.4 Model voting

We attempted to improve the diagnostic ability of the MRI interpreted by the SRs for PCa *via* model voting. We selected the diagnoses from the SVM model, RF model, ADB model, and MRI results by the SRs for voting. The analysis showed that the voting results were significantly better for the ML models than for the MRI diagnosis by the SRs (AUC: 0.72 vs. 0.89, sensitivity: 0.68 vs. 0.76, specificity: 0.77 vs. 0.90, accuracy: 0.74 vs. 0.80).

## 4 Discussion

PCa radiomics is an emerging research field with high potential to offer non-invasive and longitudinal biomarkers for personalized medicine (15). The ML-based imaging radiomics transforms visual image information into in-depth quantitative indicators, extracts a large amount of image feature information from medical images, and constructs predictive models based on feature information (16, 17). In our previous study, prostate features were extracted from TRUS videos, and the SVM model constructed using ML algorithms was found to outperform MRI-based advanced radiologist diagnosis for PCa (AUC = 0.78 vs. 0.75 in the validation set and 0.75 vs. 0.72 in the test set) (18). Techniques such as quantitative MRI analysis and computer-aided diagnosis have expanded the scope to analyze prostate MRI, and have been shown to improve diagnostic accuracy and reproducibility (19–21) and reduce inter-diagnostician variability by highlighting suspicious areas on MRI (22). In this study, we used TRUS videos and mpMRI to build ML prediction models for PCa and compared their diagnostic performance. Our analysis showed that the ML prediction model in the ADC group and the SVM model in the fusion group had the best diagnostic efficacy for PCa. The model voting results were significantly better for the ML models than for the MRI diagnosis by the SRs, indicating that the ML models can effectively assist radiologists in PCa diagnosis.

ADC can be considered as the best single-parameter component for prostate MRI assessment at present (23). In the study by Bonekamp et al., ML and imaging histology did not exceed the predictive performance of single-parameter ADC assessment (24). However, there was an overlap in the ADC values of transitional zone (TZ) PCa and stromal benign prostatic hyperplasia. Therefore, the second version of the PI-RADS suggests the use of T2W-MRI as a definitive sequence for the evaluation of the TZ (25, 26). In this study, the ML prediction models based on the mpMRI were constructed using all T2 and ADC sequence images of the prostate. Based on the sample from our previous study, our team selected the ultrasound video ML models. A total of four models were constructed by fitting the training set with GridSearchCV. The AUC in the ADC group exceeded that in the other two groups and the results obtained agreed with previous literature (23, 24).

The 3D volumetric region of interest generated was analyzed computationally, using image histology analysis software to extract a large number of image histology features. Some of the commonly extracted features were morphological, first-order, and texture features (obtained from the grayscale co-occurrence matrix, travel matrix, size region matrix, and neighborhood grayscale difference matrix). The redundancy among these large numbers of features and the fact that some of them were of minimal or no relevance to the corresponding task, necessitated feature selection and dimensionality reduction (27, 28).

Among the image histology features selected in this study (29), the BUS group had one feature from the morphological features and one from the GLSZM features for quantifying gray areas in the image; the T2 group had two from the morphological features and one from the neighborhood gray tone difference matrix features; and the ADC group had one from the morphological features, one from the first-order features (i.e., range of gray values of the ROI), one from the GLDM features, and one from the GLSZM features for quantifying gray areas in the image. In addition, the BUS, T2, and ADC groups had 18, 10, and 9 wavelet (30) subsets taken from the texture features, respectively. The predominance of wavelet-based texture features indicates that texture features have a better classification function. These features are related to the composition of heterogeneous cells in malignant tumors with significant molecular and microenvironmental differences, indicating that the texture features of tumors are highly correlated with heterogeneity (31).

In radiomics modeling, logistic regression models are the most popular and commonly used supervised classifiers owing to their simplicity and ease of implementation. Further, the commonly used ML methods include RF (32), SVM (33), and artificial neural networks. Previous literature has reported that both the SVM and RF models exhibit good stability, both models achieving good diagnostic efficiency in building small sample prediction models (34). In this study, both the BUS group and the T2 group performed best with the SVM model. Compared with the BUS group, the T2 group exhibited lower sensitivity and higher specificity and accuracy, but the AUC of the SVM model and the RF model of the BUS group were higher than those of the T2 group, indicating that the machine learning models of the two imaging sources have their own advantages and disadvantages. Previously, Li et al. compared the PCa diagnostic performance of the mpMRI imaging histology model and PI-RADS v2.1 and found that the AUC of the mpMRI imaging histology model exceeded that of PI-RADS v2.1, and that combined imaging histology could improve the diagnostic performance of PI-RADS scores (35).To evaluate the best ML prediction model for the diagnosis of PCa, we fused the features screened *via* LASSO regression from the BUS, T2, and ADC groups and re-used the SVM, RF, ADB, and GBM models for another comprehensive feature model comparison in this study. The results showed that the combined features (BUS+ADC+T2) had better efficacy in the diagnosis of PCa relative to ADC, while the SVM model was superior to the other models in terms of diagnostic efficacy. Since the GBM model was less effective than the other three models, we selected the SVM model, RF model, ADB model, and MRI diagnosis by the SRs for voting. The analysis showed that the combined voting results were significantly better for the ML models than for the MRI diagnosis by the SRs (AUC: 0.89 vs. 0.72, sensitivity: 0.76 vs. 0.68, specificity: 0.90 vs. 0.77, accuracy: 0.80 vs. 0.74), indicating that the ML integrated models can effectively assist radiologists in the diagnosis of PCa.

The ML models constructed on the basis of TRUS videos and mpMRI improved the diagnosis of PCa but did not guarantee the utility of accurately ruling each other out. A fusion model of the two techniques provides complementary information that is expected to become more important in the era of focal therapy to accurately identify the location of PCa.

There are several limitations to this study. First, the study was conducted at a single center, and we therefore cannot rule out a single-center effect. Second, since this was a retrospective study with a small sample size, and the findings were only preliminary, multi-center and large-sample studies are still needed to test the validation set and prospective studies to verify the reliability of the models. Third, only the value of the T2 sequences from TRUS videos and mpMRI with ADC sequences in predicting PCa was investigated. The predictive value of the comparison between the ML models using ADC images with b-values and ADC images combined with clinical features was not considered, since it was not the focus of this study. Further integration of clinical and imaging histology features to construct models will be performed in later studies. We aim to build a robust prediction model to replace other imaging methods and provide a solid theoretical basis for accurate and individualized treatment of PCa.

## 5 Conclusions

In our study, we used ultrasound videos, mpMRI T2 sequences, and ADC sequences to form separate datasets to build ML models. The prediction models constructed using ML algorithms showed a good diagnostic ability for PCa. The accuracy, sensitivity, and specificity of the SVM model in the ADC group were better than those in the BUS and T2 groups. The SVM model in the fusion group showed the best diagnostic performance for PCa. The model showed a better diagnostic efficacy in the fusion group than in the ADC, BUS, and T2 groups in both the validation and test cohorts. In the fusion group, the SVM model showed a better performance than did the RF model. These prediction models can help radiologists make better diagnoses. In our future work, we plan to combine the comparison outcomes of the ML models based on ADC images of prostate MRI and ADC b-values with clinical features to build a better ML model and use deep learning and neural networks for ultrasound diagnosis of PCa. The models established in this study can help in achieving more accurate diagnoses and differential diagnoses of lesions, which can greatly aid in clinical treatment decision-making and prognosis prediction.

## Data availability statement

The raw data supporting the conclusions of this article will be made available by the authors, without undue reservation.

## Ethics statement

The studies involving human participants were reviewed and approved by Medical Ethics Committee of Dongyang Hospital of Wenzhou Medical University. The ethics committee waived the requirement of written informed consent for participation.

## Author contributions

Conceptualization, XQ, KW, LZ and JCY. Methodology, XQ,KW, ZW and BF. Software, BF, JCY and KW. Validation, LZ, ZW and JCY. Formal analysis, XQ and KW. investigation, XQ, KW and BF. Resources, LZ, ZW and JCY. Data curation, XS, JY, ZH, MZ, CL and LJ. Writing—original draft preparation, XQ, KW and BF. Writing—review and editing, LZ, ZW and JCY. Visualization, LZ, ZW and JY. Supervision, LZ, ZW and JCY. Project administration, LZ, ZW and JCY. Funding acquisition, MZ, KW. All authors have read and agreed to the published version of the manuscript.

## References

[1] Stabile A Dell'Oglio P Soligo M De Cobelli F Gandaglia G Fossati N. Assessing the clinical value of positive multiparametric magnetic resonance imaging in young men with a suspicion of prostate cancer. Eur Urol Oncol (2021) 4(4):594–600. doi: 10.1016/j.euo.2019.05.006 31204312

[2] Culp MB Soerjomataram I Efstathiou JA Bray F Jemal A. Recent global patterns in prostate cancer incidence and mortality rates. Eur Urol (2020) 77(1):38–52. doi: 10.1016/j.eururo.2019.08.005 31493960

[3] Bray F Ferlay J Soerjomataram I Siegel RL Torre LA Jemal A. Global cancer statistics 2018: GLOBOCAN estimates of incidence and mortality worldwide for 36 cancers in 185 countries. CA Cancer J Clin (2018) 68(6):394–424. doi: 10.3322/caac.21492 30207593

[4] Ilic D Djulbegovic M Jung JH Hwang EC Zhou Q Cleves A. Prostate cancer screening with prostate-specific antigen (PSA) test: a systematic review and meta-analysis. BMJ (2018) 362:k3519. doi: 10.1136/bmj.k3519 30185521 PMC6283370

[5] Vale CL Fisher D Kneebone A Parker C Pearse M Richaud P. Adjuvant or early salvage radiotherapy for the treatment of localised and locally advanced prostate cancer: a prospectively planned systematic review and meta-analysis of aggregate data. Lancet (2020) 396(10260):1422–31. doi: 10.1016/S0140-6736(20)31952-8 PMC7611137 33002431

[6] Mottet N van den Bergh RCN Briers E Van den Broeck T Cumberbatch MG De Santis M. EAU-EANM-ESTRO-ESUR-SIOG guidelines on prostate cancer-2020 update. part 1: screening, diagnosis, and local treatment with curative intent. Eur Urol (2021) 79(2):243–62. doi: 10.1016/j.eururo.2020.09.042 33172724

[7] Philip J Manikandan R Javlé P Foster CS. Prostate cancer diagnosis: should patients with prostate specific antigen >10ng/mL have stratified prostate biopsy protocols? Cancer Detect Prev (2009) 32(4):314–8. doi: 10.1016/j.cdp.2008.12.004 19193497

[8] Ozorak A Zumrutbas AE Bingol G Ozlulerden Y Ozturk SA. Prostate cancer incidence and diagnosis in men with PSA levels >20 ng/ml: is it possible to decrease the number of biopsy cores? Aging Male (2020) 23(5):893–900. doi: 10.1080/13685538.2019.1620204 31156017

[9] Feng L Shi L Jiang YM. Clinical value of transrectal ultrasound combined with prostate specific antigen density in the diagnosis of prostate cancer. Imaging Sci Photochem (2020) 38(2):323–7. doi: 10.7517/issn.1674-0475.190925

[10] Würnschimmel C Chandrasekar T Hahn L Esen T Shariat SF Tilki D. MRI As a screening tool for prostate cancer: current evidence and future challenges. World J Urol (2022) 41(4):921–928. doi: 10.1007/s00345-022-03947-y PMC10160206 35226140

[11] Westphalen AC McCulloch CE Anaokar JM Arora S Barashi NS Barentsz JO. Variability of the positive predictive value of PI-RADS for prostate MRI across 26 centers: experience of the society of abdominal radiology prostate cancer disease-focused panel. Radiology. (2020) 296(1):76–84. doi: 10.1148/radiol.2020190646 32315265 PMC7373346

[12] Zhang JH Xu LL Zhang GM Sun H Jin ZY. [Research progress of magnetic resonance imaging-based radiomics in prostate cancer]. Zhongguo Yi Xue Ke Xue Yuan Xue Bao (2022) 44(1):123–9. doi: 10.3881/j.issn.1000-503X.14075 35300774

[13] van Griethuysen JJM Fedorov A Parmar C Hosny A Aucoin N Narayan V. Computational radiomics system to decode the radiographic phenotype. Cancer Res (2017) 77(21):e104–7. doi: 10.1158/0008-5472.CAN-17-0339 PMC5672828 29092951

[14] Pedregosa F Varoquaux G Gramfort A Michel V Thirion B Grisel O. Scikit-learn: machine learning in Python. J Mach Learn Res (2011) 12:2825–30. doi: 10.5555/1953048.2078195

[15] Spohn SKB Bettermann AS Bamberg F Benndorf M Mix M Nicolay NH. Radiomics in prostate cancer imaging for a personalized treatment approach-current aspects of methodology and a systematic review on validated studies. Theranostics (2021) 11(16):8027–42. doi: 10.7150/thno.61207 PMC8315055 34335978

[16] Mayerhoefer ME Materka A Langs G Häggström I Szczypiński P Gibbs P. Introduction to radiomics. J Nucl Med (2020) 61(4):488–95. doi: 10.2967/jnumed.118.222893 PMC9374044 32060219

[17] Avanzo M Stancanello J Pirrone G Sartor G. Radiomics and deep learning in lung cancer. Strahlenther Onkol (2020) 196(10):879–87. doi: 10.1007/s00066-020-01625-9 32367456

[18] Wang K Chen P Feng B Tu J Hu Z Zhang M. Machine learning prediction of prostate cancer from transrectal ultrasound video clips. Front Oncol (2022) 12:948662. doi: 10.3389/fonc.2022.948662 36091110 PMC9459141

[19] Shinmoto H Tamura C Soga S Shiomi E Yoshihara N Kaji T. An intravoxel incoherent motion diffusion-weighted imaging study of prostate cancer. AJR Am J Roentgenol (2012) 199(4):W496–500. doi: 10.2214/AJR.11.8347 22997399

[20] Tamura C Shinmoto H Soga S Okamura T Sato H Okuaki T. Diffusion kurtosis imaging study of prostate cancer: preliminary findings. J Magn Reson Imaging (2014) 40(3):723–9. doi: 10.1002/jmri.24379 24924835

[21] Fei B. Computer-aided diagnosis of prostate cancer with MRI. Curr Opin BioMed Eng (2017) 3:20–7. doi: 10.1016/j.cobme.2017.09.009 PMC5931723 29732440

[22] Greer MD Lay N Shih JH Barrett T Bittencourt LK Borofsky S. Computer-aided diagnosis prior to conventional interpretation of prostate mpMRI: an international multi-reader study. Eur Radiol (2018) 28(10):4407–17. doi: 10.1007/s00330-018-5374-6 PMC8023433 29651763

[23] Chatterjee A Watson G Myint E Sved P McEntee M Bourne R. Changes in epithelium, stroma, and lumen space correlate more strongly with Gleason pattern and are stronger predictors of prostate ADC changes than cellularity metrics. Radiology (2015) 277(3):751–62. doi: 10.1148/radiol.2015142414 26110669

[24] Bonekamp D Kohl S Wiesenfarth M Schelb P Radtke JP Götz M. Radiomic machine learning for characterization of prostate lesions with MRI: comparison to ADC values. Radiology (2018) 289(1):128–37. doi: 10.1148/radiol.2018173064 30063191

[25] Barentsz JO Weinreb JC Verma S Thoeny HC Tempany CM Shtern F. Synopsis of the PI-RADS v2 guidelines for multiparametric prostate magnetic resonance imaging and recommendations for use. Eur Urol (2016) 69(1):41–9. doi: 10.1016/j.eururo.2015.08.038 PMC6364687 26361169

[26] Weinreb JC Barentsz JO Choyke PL Cornud F Haider MA Macura KJ. PI-RADS prostate imaging - reporting and data system: 2015, version 2. Eur Urol (2016) 69(1):16–40. doi: 10.1016/j.eururo.2015.08.052 26427566 PMC6467207

[27] Kumar V Gu Y Basu S Berglund A Eschrich SA Schabath MB. Radiomics: the process and the challenges. Magn Reson Imaging (2012) 30(9):1234–48. doi: 10.1016/j.mri.2012.06.010 PMC3563280 22898692

[28] Yip SS Aerts HJ. Applications and limitations of radiomics. Phys Med Biol (2016) 61(13):R150–66. doi: 10.1088/0031-9155/61/13/R150 PMC4927328 27269645

[29] Miotto R Wang F Wang S Jiang X Dudley JT. Deep learning for healthcare: review, opportunities and challenges. Brief Bioinform (2018) 19(6):1236–46. doi: 10.1093/bib/bbx044 PMC6455466 28481991

[30] de Cheveigné A Nelken I. Filters: when, why, and how (Not) to use them. Neuron (2019) 102(2):280–93. doi: 10.1016/j.neuron.2019.02.039 30998899

[31] Jin X Ai Y Zhang J Zhu H Jin J Teng Y. Noninvasive prediction of lymph node status for patients with early-stage cervical cancer based on radiomics features from ultrasound images. Eur Radiol (2020) 30(7):4117–24. doi: 10.1007/s00330-020-06692-1 32078013

[32] Chen T Li M Gu Y Zhang Y Yang S Wei C. Prostate cancer differentiation and aggressiveness: assessment with a radiomic-based model vs. PI-RADS v2. J Magn Reson Imaging (2019) 49(3):875–84. doi: 10.1002/jmri.26243 PMC6620601 30230108

[33] Shiradkar R Ghose S Jambor I Taimen P Ettala O Purysko AS. Radiomic features from pretreatment biparametric MRI predict prostate cancer biochemical recurrence: preliminary findings. J Magn Reson Imaging (2018) 48(6):1626–36. doi: 10.1002/jmri.26178 PMC6222024 29734484

[34] Li W Huang Y Zhuang BW Liu GJ Hu HT Li X. Multiparametric ultrasomics of significant liver fibrosis: a machine learning-based analysis. Eur Radiol (2019) 29(3):1496–506. doi: 10.1007/s00330-018-5680-z PMC6510867 30178143

[35] Li M Chen T Zhao W Wei C Li X Duan S. Radiomics prediction model for the improved diagnosis of clinically significant prostate cancer on biparametric MRI. Quant Imaging Med Surg (2020) 10(2):368–79. doi: 10.21037/qims.2019.12.06 PMC7063275 32190563

